# Large emission mitigation potential from sequencing fossil fuel extraction infrastructure retirement in China

**DOI:** 10.1016/j.isci.2026.117563

**Published:** 2026-09-18

**Authors:** Yi Jin, Xiaoyang Zhong, Arnold Tukker, Xu Tang, Elina Brutschin, Zhida Ma, Yingchao Chen, Wenchao Li, Lixin Tian, Bas van Ruijven, Paul Behrens

**Affiliations:** 1 Institute of Industrial Economics, School of Finance and Economics, Jiangsu University, Zhenjiang, Jiangsu 212013, China; 2 Institute of Environment and Ecology, Tsinghua Shenzhen International Graduate School, Tsinghua University, Shenzhen, China; 3 Energy, Climate, and Environment (ECE) Program, International Institute for Applied Systems Analysis (IIASA), Laxenburg, Austria; 4 Institute of Environmental Sciences (CML), Leiden University, Leiden 2333 CC, the Netherlands; 5 Netherlands Organisation for Applied Scientific Research TNO, den Haag, the Netherlands; 6 School of Economics and Management, China University of Petroleum (Beijing), Beijing 102249, China; 7 School of Economics, Shandong Technology and Business University, Yantai, Shandong 255000, China; 8 School of Mathematical Sciences, Jiangsu University, Zhenjiang, Jiangsu 212013, China; 9 Oxford Martin School, University of Oxford, Oxford, UK

**Keywords:** fossil fuel extraction, retirement sequencing, carbon mitigation, asset-level analysis, China, energy transition

## Abstract

China’s fossil fuel infrastructure could lock in substantial emissions across both extraction and downstream use, making a well-defined phaseout strategy urgent for achieving carbon neutrality. We analyze the differences between the current retirement strategy and the proposed emission-oriented retirement sequencing strategy by quantifying the emission mitigation effect, employment benefit, and cost-saving advantage of strategies. We show that China’s fossil fuel emissions totaled 9,850 MtCO_2_e in 2023 and are projected to reach 98.9–148.5 GtCO_2_e cumulatively during 2024–2050 under existing plans across energy-demand scenarios. Strategically sequencing retirements could avoid a cumulative 3.8–4.8 GtCO_2_e by 2050, more than annual European emissions, while simultaneously increasing employment by 4–6 million full-time equivalent-years and reducing net total system costs by 245–507 billion CNY (around 34–71 billion USD). These results highlight the climate, economic, and social benefits of sequencing-based phaseout strategies.

## Highlights

• A China’s fossil fuel extraction infrastructure inventory is compiled
• Emission intensities vary substantially across infrastructure types
• Shifting retirement strategy could avoid a cumulative 3.8–4.8 GtCO_2_e by 2050
• Shifting retirement strategy could result in employment increase and cost savings

## Graphical abstract

**Figure:**
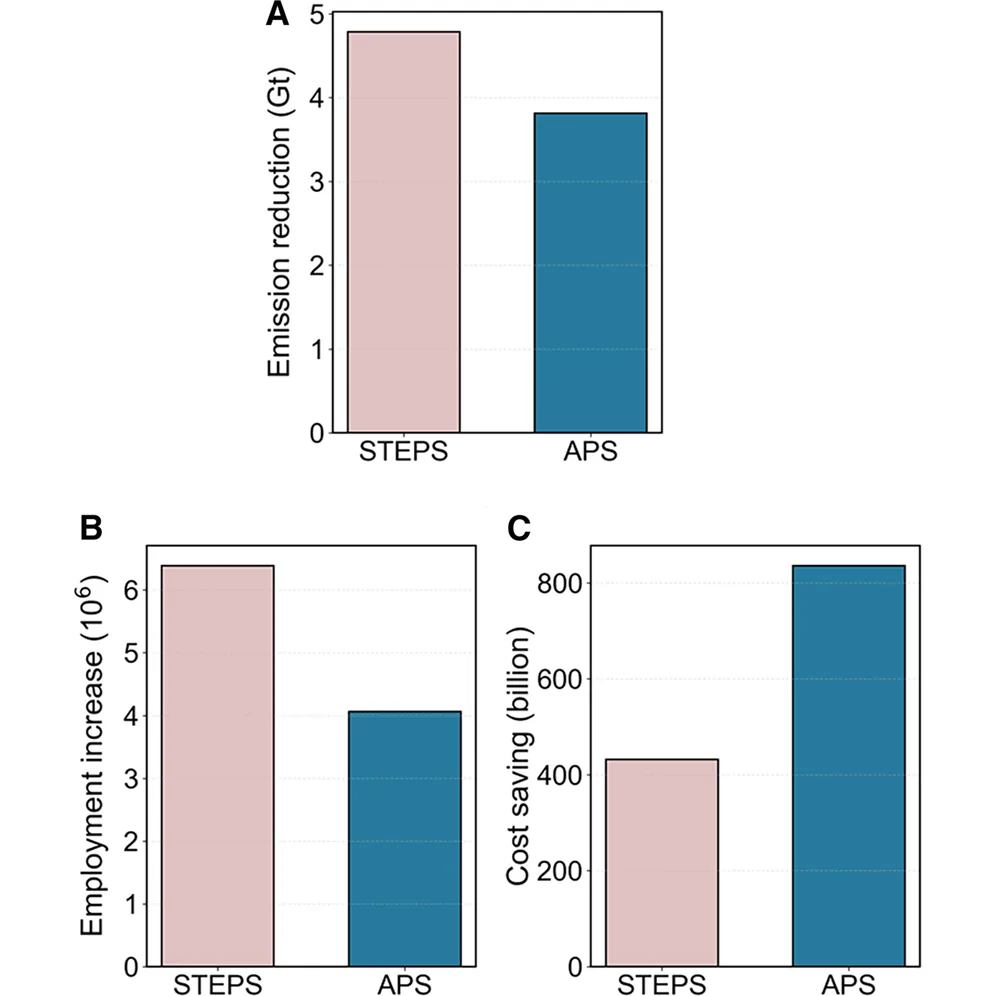

## Teaser

Environmental science; Applied sciences

## Introduction

Meeting global climate agreements for a 1.5°C scenario requires a halt on all new fossil fuel infrastructure.^1^ On top of this, a rapid and well-ordered retirement of fossil fuel assets is essential for mitigating emissions, addressing climate change, and ensuring energy supply.^2^^,^^3^^,^^4^ Despite some positive signs in the speed of the energy transition around the world, some countries, especially large fossil fuel producers and consumers, such as China, have not yet seen any sustained reduction in fossil use.^5^^,^^6^^,^^7^ Between 2019 and 2024, China’s gas, coal, and oil use have continued to grow at annual rates of 6.5%, 4.5%, and 1.8%, respectively.^8^

Studies suggest that China’s emissions may peak and decline in the coming years with a phaseout of fossil fuels and renewable energy transition.^9^^,^^10^ The sequencing by which these fossil assets are retired can impact cumulative emissions throughout the transition.^11^ Prioritizing retirement of infrastructure with higher emissions over infrastructure with lower emissions with the same energy output would reduce emissions faster and limit warming.^12^^,^^13^

Much of the literature has focused on the emissions from fuel combustion in end-use and power sectors,^14^^,^^15^^,^^16^^,^^17^^,^^18^ while the emissions implications of retiring fossil fuel extraction infrastructure such as mines remain understudied. Extraction shapes system-wide emissions in two ways: (1) direct, on-site emissions from energy use and fugitive releases, and (2) downstream emissions associated with the combustion of the fuels supplied, which are determined by the asset-specific carbon content and heating value.^16^^,^^19^^,^^20^^,^^21^ Prior environmental impact assessment tools widely adopted in energy research include life-cycle assessment (LCA),^22^ multi-regional input-output (MRIO) analysis,^23^ and material flow analysis (MFA).^24^ Traditional LCA quantifies carbon footprints of energy assets, MRIO captures interregional carbon transfer embodied in fossil trade, and MFA tracks the carbon flows in fossil industrial chains. Few studies combine site-level impact accounting with dynamic asset retirement simulation to compare differentiated retirement strategies for extraction infrastructure.

This work integrates site-level impact assessment, incorporates energy supply demand characteristics, and expands conventional single-indicator evaluation into a multi-indicator dynamic simulation under various scenarios and retirement strategies, which enriches the methodological toolkit for fossil-fuel impact assessment. Specifically, we provide the first asset-level estimate of emissions across both extraction and downstream use for China’s fossil fuel infrastructure. We then assess how retirement sequences for extraction infrastructure reshape cumulative emissions across energy-transition scenarios, covering both direct extraction and downstream fuel-use emissions. After identifying the least-emission strategy, we assess its implications for employment and extraction costs to explore potential tradeoffs. We do this for a large inventory of fossil fuel sites (coal mines, oil fields, and gas fields) with information on location, output, remaining reserves, on-site and combustion emission intensities, on-site employment, costs, and more. We cover 81%, 82%, and 89% of the national output of coal, gas, and oil, respectively (see STAR Methods for more).

This study presents three key innovations. First, unlike existing research focusing on downstream fuel combustion, we build a high-coverage micro-inventory of coal, oil, and gas extraction sites and realize unified emission accounting covering both extraction and combustion emissions at the asset level. Second, against the prevailing small-site-first retirement strategy, we develop an emission-oriented retirement sequencing strategy and assess its impacts under energy demand scenarios. Third, beyond carbon emission assessment in prior supply side studies, we establish a triple-dimension framework integrating emission mitigation, employment retention, and cost savings, filling the gap of multi-objective assessment for China’s upstream fossil infrastructure retirement.

We find that China’s fossil fuel extraction infrastructure emitted 9.85 GtCO_2_e in 2023 and is on track to generate 98.9–148.5 GtCO_2_e between 2024 and 2050 under current retirement plans. We show that the emission-oriented retirement of emission-intensive sites—closing the most emission-intensive sites first—rather than the current practice of prioritizing smaller sites—could avoid a cumulative 3.8–4.8 GtCO_2_e during 2024–2050, comparable to the annual emissions of the European continent. Such sequencing would also soften socio-economic impacts, preserving 4–6 million full-time-equivalent job-years and reducing extraction costs by CNY 245–507 billion (USD 34–71 billion). Realizing these benefits would require coordinated national planning, better asset-level data, and active engagement with local stakeholders and asset owners.

## Results

### Current fossil-fuel emissions, employment, and cost

China’s fossil fuel emissions across extraction and downstream use totaled 9,850 MtCO_2_e in 2023, with coal, oil, and gas accounting for 89%, 6%, and 5% of the total, respectively. Of these emissions, 85% resulted from fuel combustion and 15% from extraction. Emissions were unevenly distributed across the country (Figure 1), with Shanxi (2,581 MtCO_2_e), Inner Mongolia (2,389 MtCO_2_e), and Shaanxi (1,658 MtCO_2_e) emerging as the top emitters. Total unburned emissions for the remaining fossil reserves across China are 597 GtCO_2_e—around 53 times China’s projected emissions in 2024 (see the Data S1 for all site-level emissions). China’s fossil fuel employment totaled 2.6 million, with coal, oil, and gas accounting for 59%, 18%, and 23% of the total, respectively. China’s fossil fuel cost totaled CNY 1,456 billion, with coal, oil, and gas accounting for 68%, 18%, and 14% of the total, respectively.

**Figure 1:**
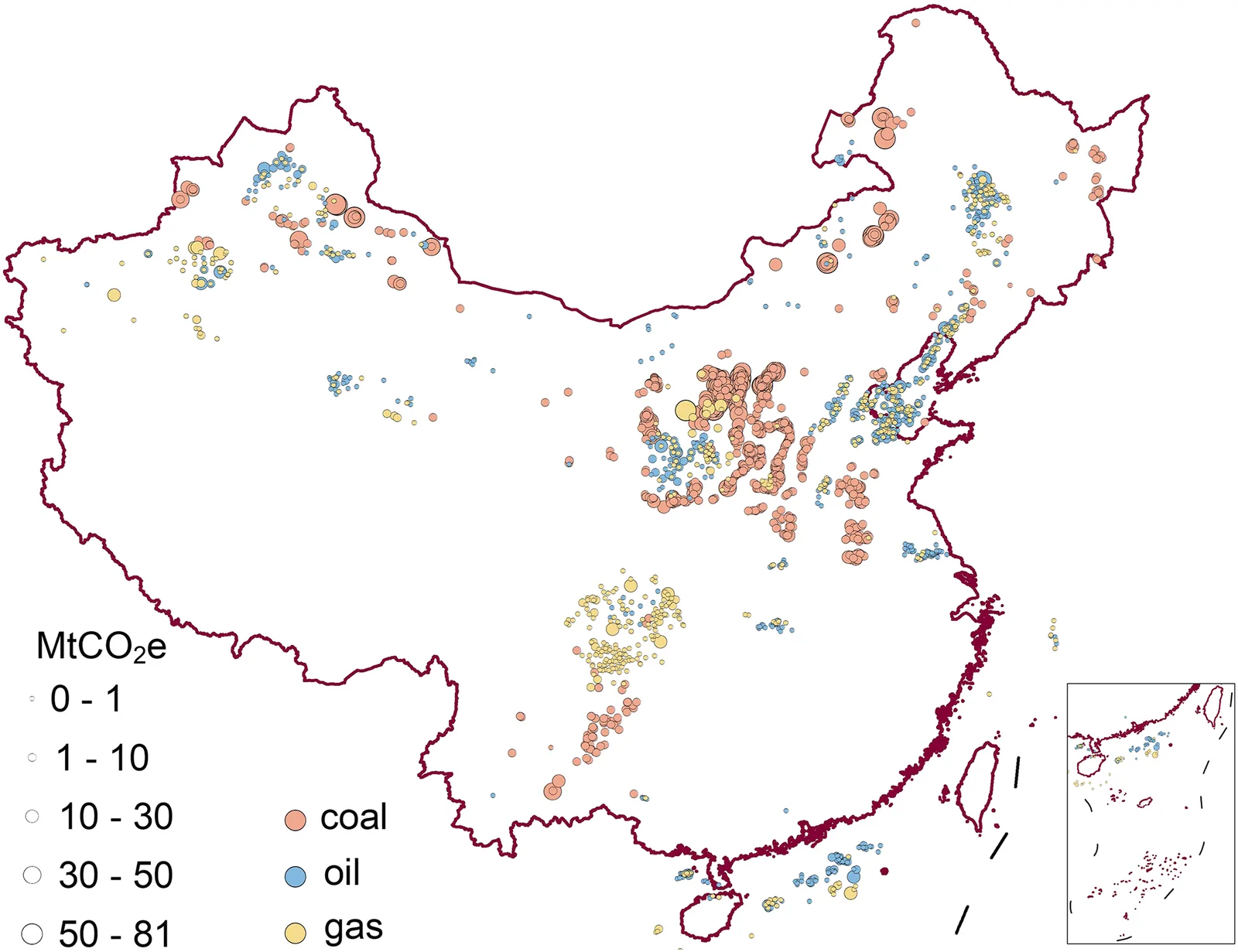
Current emissions from the annual fuel supply Emissions are expressed as CO_2_-equivalents (CO_2_e; GWP100) and include extraction and subsequent combustion (STAR Methods). Dots represent 2693 coal, oil, and gas extraction infrastructures.

Yet not all fossil fuel sites contribute equally to this potential burden. The emissions differ markedly across infrastructures, with top 206 out of 2,693 energy production sites accounting for 50% of total emissions. The site-level emissions see a wide range from 34 tCO_2_e to 81 MtCO_2_e (Figure 2A). Emission intensities vary widely across sites and do not correlate with their output (Figure 2B). This variation is driven primarily by the carbon content of the fossil fuels, to the detriment of combustion emissions, rather than site scale. Employment intensity and extraction cost also differ across fossil fuel sites with implications for the socio-economic impacts of fossil phase-out. Smaller sites across all fossil fuel types generally have higher employment intensity and smaller coal sites generally have a higher extraction cost than larger sites (see Data S1 for details of site-level employment and costs).

**Figure 2:**
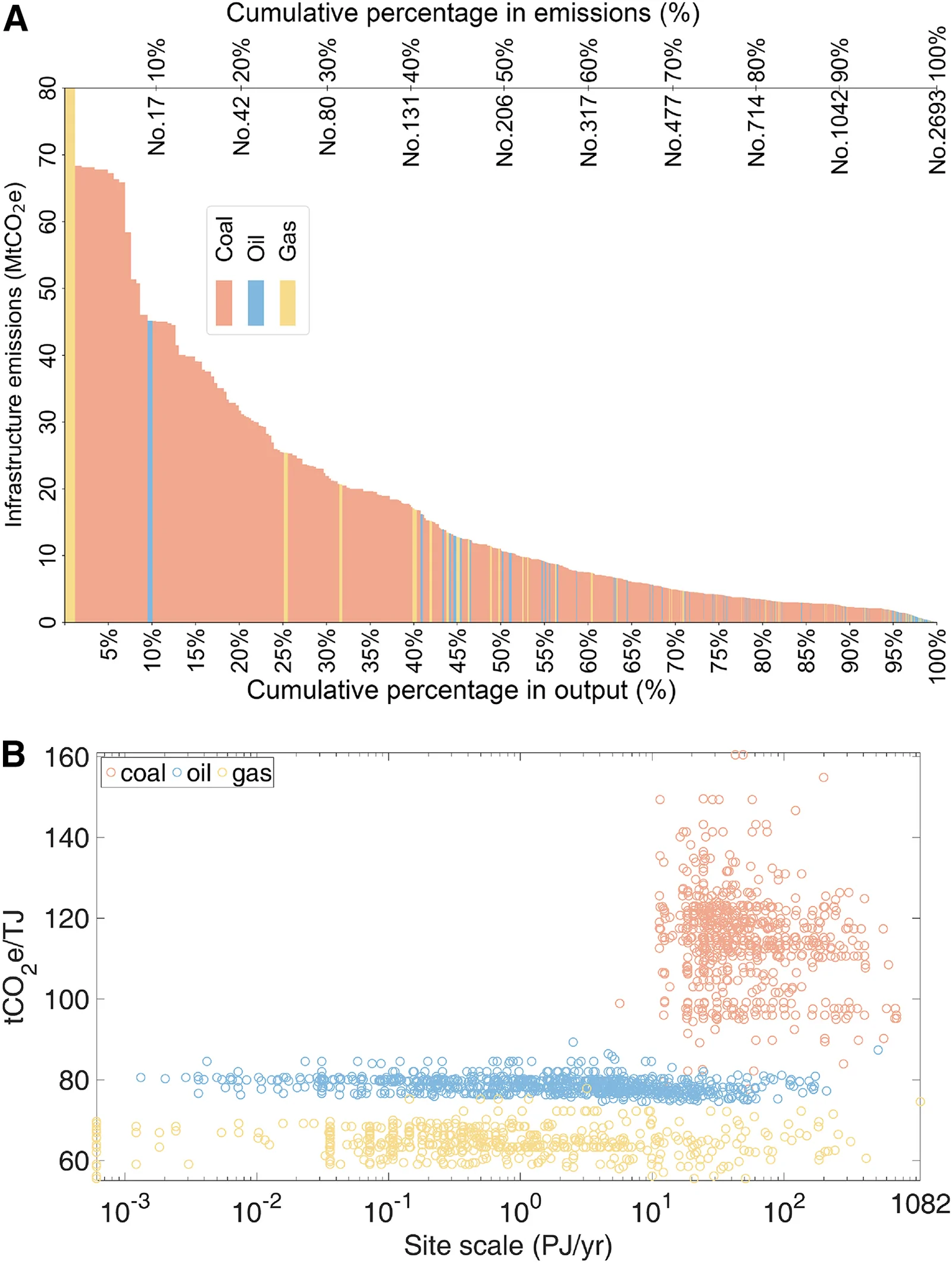
Current emissions from the annual fuel supply (A) Emissions of extraction and combustion in descending order for 2,693 coal, oil, and gas extraction infrastructures. (B) Emission intensities versus site scale.

### Retirement strategies and future emissions, employment, and cost

Given these dynamics, focusing solely on scale in retirement strategies means missing out on critical mitigation opportunities. Yet, current policies across China prioritize shutting down smaller sites.^25^ We examine emissions under this current priority (which we name *current*) and under an alternative strategy which prioritizes high-emission-intensity sites while still maintaining security of supply (which we name *emission-oriented*) (Table 1). That is, we optimize only on the basis of lowering emissions. We combine these two strategies with two energy demand scenarios: the International Energy Agency’s (IEA) Stated Policies Scenario (STEPS), reflecting current expectations, and the Announced Pledges Scenario (APS), where all national energy and climate targets made by governments are met in full and on time (Figure 3).^26^ Since the two retirement strategies—current and emission-oriented—do not alter the quantity of fossil fuels consumed, they differ only in which extraction assets supply that fixed demand. Consequently, the emission differences reported between strategies reflect the heterogeneity in asset-level emission intensities across the portfolio. On top of these targets, we assume fossil fuel sites will also shut down due to the depletion of reserves (in the case of fuel extraction sites) and economic infeasibility (driven by an increasing carbon price) (Figure 4). We calculate annual emissions from operating infrastructure during 2024–2050. Finally, we explore the employment and cost impacts of these different strategies.

**Table 1:** Energy scenarios and drivers of fossil fuel retirement in this study

Energy scenarios\Drivers of closure	STEPS	APS
Reserve depletion	reserve depletion results in site closure, which is irrelevant to energy demand scenarios and other closure drivers (economic infeasibility and closure strategies)	reserve depletion results in site closure, which is irrelevant to energy demand scenarios and other closure drivers (economic infeasibility and closure strategies)
Economically infeasibility	economic infeasibility refers to fossil fuel sites stranded by carbon price; the carbon price is lower in STEPS than in APS	economic infeasibility refers to fossil fuel sites stranded by carbon price; the carbon price is higher in APS than in STEPS
Retirement strategies	*current* strategy: prioritizes small-scale sites *emission-oriented* strategy: prioritizes high-emission-intensity sites	*current* strategy: prioritizes small-scale sites *emission-oriented* strategy: prioritizes high-emission-intensity sites

**Figure 3:**
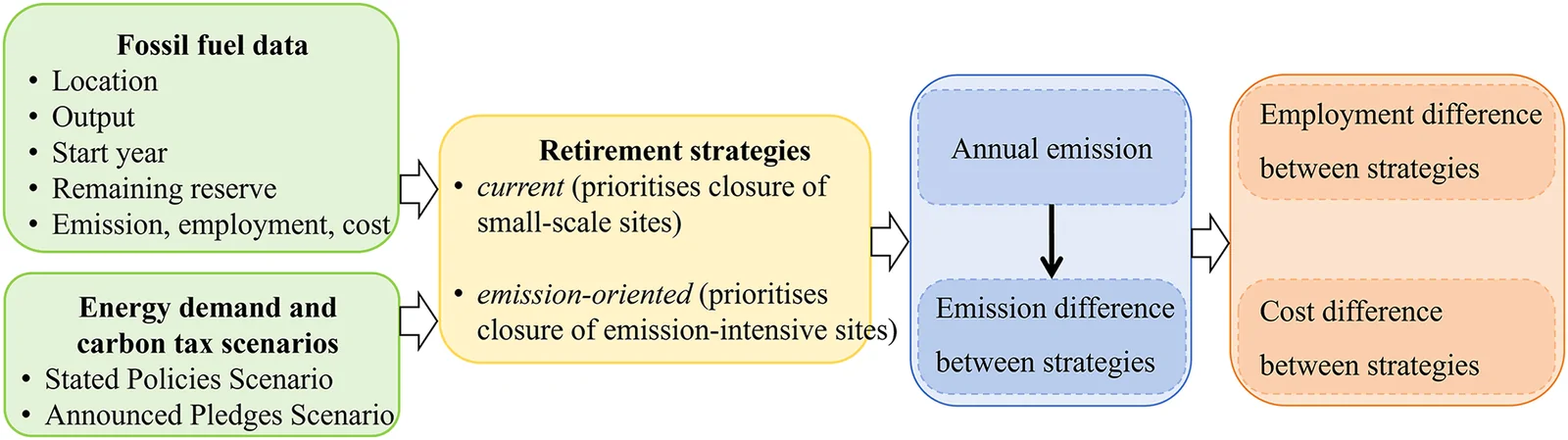
Overview of the analytical workflow Energy and carbon price components are in green, retirement strategies in yellow, emission assessment in blue, and employment and cost assessment in orange.

**Figure 4:**
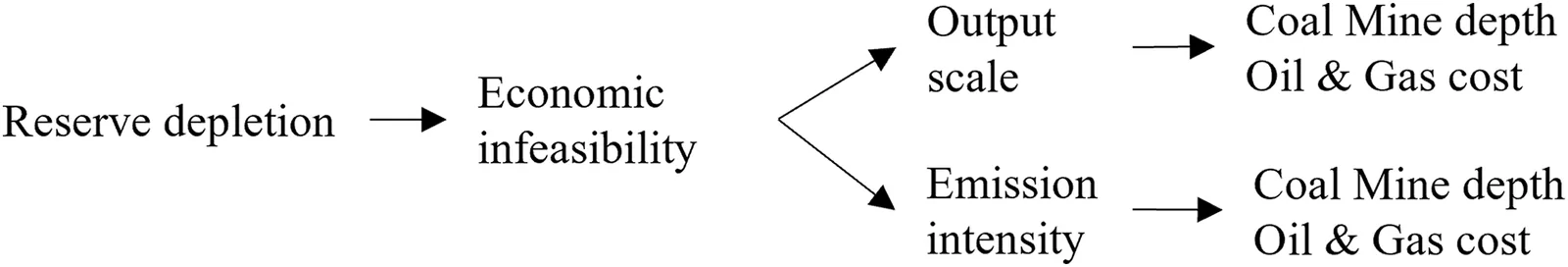
The flow for determining the sequence of fossil-fuel-site closure Two regulation strategies are set, with one prioritizing small-scale sites and another prioritizing high-emission-intensity sites.

We assess the change in annual emissions when switching from the *current* to the *emission-oriented* strategy (Figure 5) All scenarios see a rapid emission decline over 2024–2050. Annual emissions would decrease to 2,216 MtCO_2_e and 2,058 MtCO_2_e in 2050 under STEPS-*current* and STEPS-*emission-oriented*, respectively, and decrease to just 205 MtCO_2_e in 2050 under both APS-*current* and APS-*emission-oriented* scenarios. The cumulative emissions are 148.5 GtCO_2_e and 143.7 GtCO_2_e for STEPS-*current* and STEPS-*emission-oriented*, respectively, and are 102.8 GtCO_2_e and 98.9 GtCO_2_e for APS-*current* and APS-*emission-oriented*, respectively. Overall, the closure of higher emission-intensity sites (*emission-oriented* strategy) results in a large reduction in cumulative emissions (3.8–4.8 GtCO_2_e, depending on the energy demand scenario). This is equivalent to the annual emissions of the European continent.^26^

**Figure 5:**
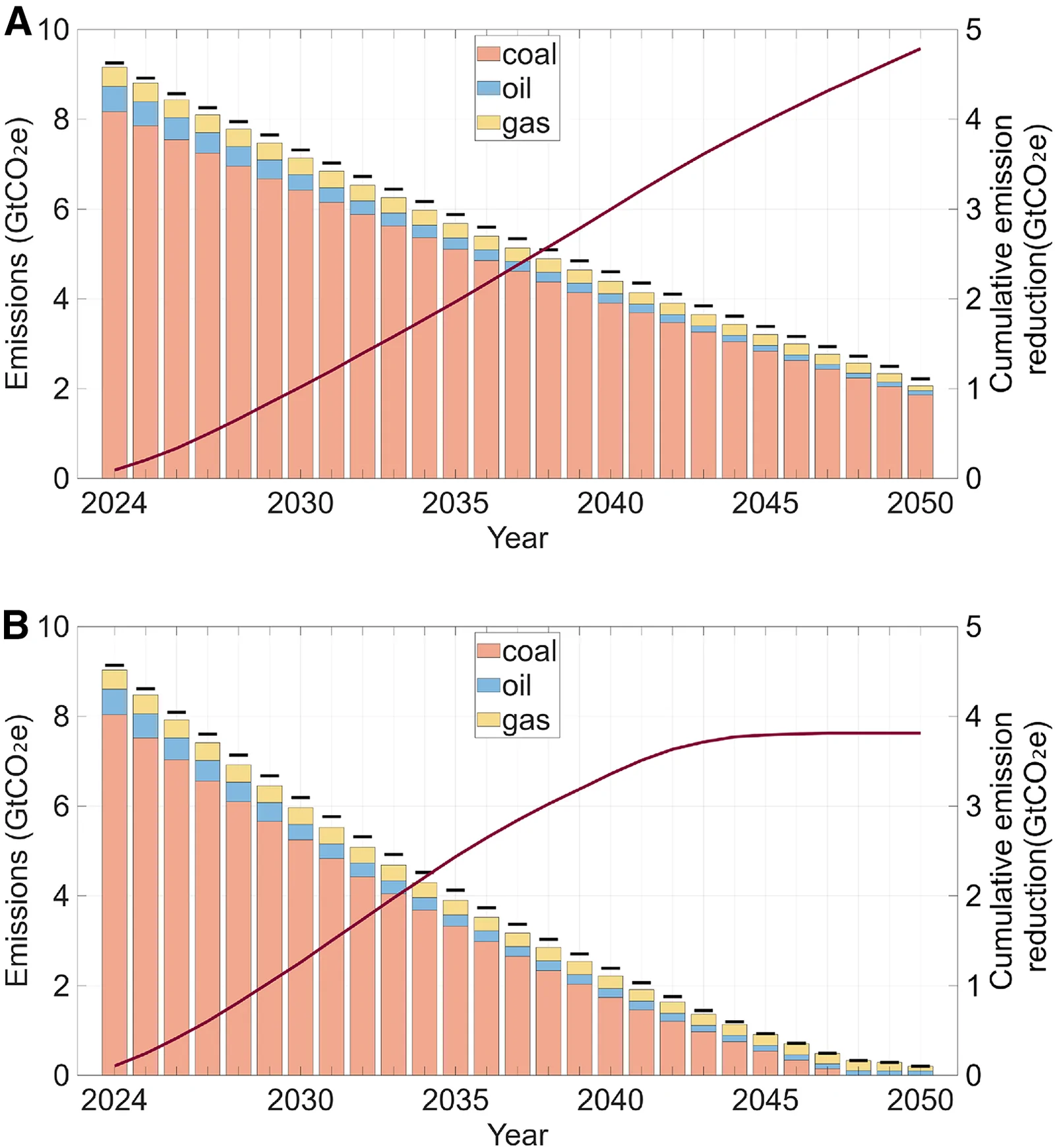
Future annual emissions during 2024-2050 Annual emissions (2024–2050) for scenario STEPS (A) and APS (B). The bars (left *y* axis) represent the emissions from coal, oil, and gas under the strategy *emission-oriented*; short black lines above each bar represent the emissions under the strategy *current*. The dark-red line (right *y* axis) represents the cumulative emission reduction under the strategy *emission-oriented* in relative to the strategy *current*. Emissions are expressed as CO_2_-equivalents (CO_2_e; GWP100) and include extraction and subsequent combustion (STAR Methods).

We also find that the *emission-oriented* strategy could support more employment than the *current* strategy. Between 2024 and 2050, it could maintain 6.4 and 4.1 million full-time equivalent-years more than the *current* strategy under the STEPS and APS scenarios, respectively—equivalent to 1.5–2.5 times the current fossil fuel workforce (Figure 6A).

**Figure 6:**
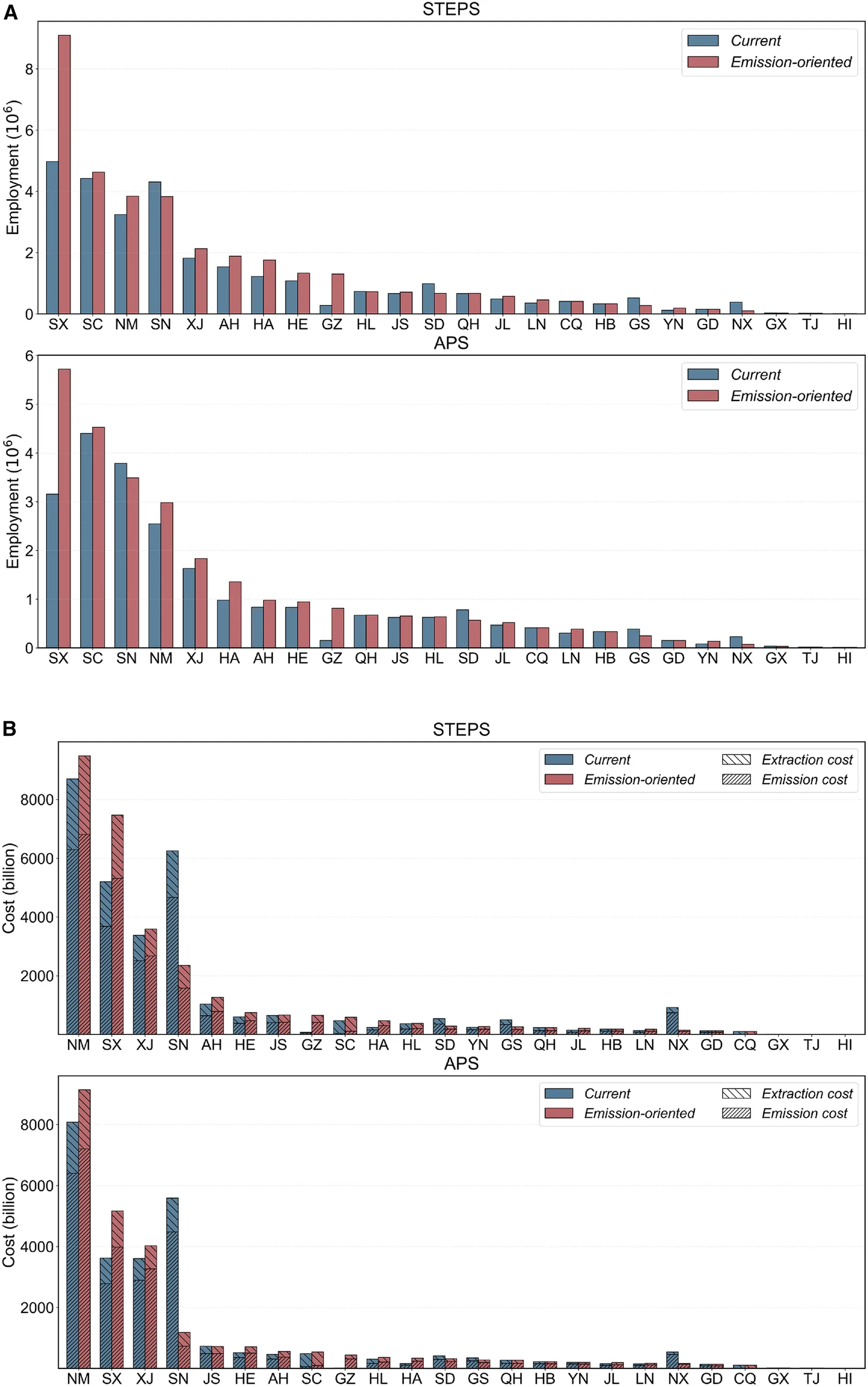
Provincial-level cumulative employment and cost under the strategy *current* and *emission-oriented* for scenario STEPS and APS over 2030–2050 (A) Employment. (B) Extraction and emission cost.

The *emission-oriented* strategy leads to a lower cost of fossil fuel extraction than the *current* strategy. During 2030–2050, CNY 432 billion and CNY 836 billion (net present value with 5% discount rate) could be saved by switching from the *current* strategy to the *emission-oriented* strategy for STEPS and APS, respectively (Figure 6B). These savings are driven by lower carbon price payments under the *emission-oriented* strategy—CNY 806 billion (STEPS) and CNY 1073 billion (APS), reflecting reduced emissions—which more than offset higher on-site extraction expenditures of CNY 374 billion and CNY 237 billion, respectively, as production shifts toward smaller, typically higher-cost sites (STAR Methods). The larger cost savings under APS are mainly due to higher carbon price. We further present provincial cumulative employment and decomposed cost from 2030 to 2050 under the current and emission-oriented retirement strategies for STEPS and APS scenarios. Under both scenarios, the emission-oriented sequencing sustains larger workforces in fossil production bases including Shanxi and Inner Mongolia, yet reduces cumulative employment in provincial regions such as Shaanxi and Sichuan. Emission-oriented strategy delivers cuts in emission cost (determined by emission quantity and carbon price) for provinces such as Shaanxi. Provinces (e.g., Shanxi) that experience more fossil production under emission-oriented strategy would generally see higher extraction cost while those (e.g., Shaanxi) with reduced fossil production would see lower extraction cost. Some provinces see negligible difference in employment (e.g., Qinghai) and cost (e.g., Jiangsu) under the current and emission-oriented retirement strategies.

We further resolve the micro-mechanisms driving employment and cost shifts by stratifying outcomes by fuel category and facility scale (Figure S2). Consistent with the inherent property that small extraction units carry higher employment intensity and extraction cost, emission-oriented sequencing delivers sustained employment retention and cost saving primarily via the retention of some low-emission small infrastructure, which might be eliminated first under current strategy. Coal infrastructure dominate employment and cost disparities between strategies, as coal mines see noticeable differences in retirement sequencing between the two strategies, while oil and gas does not see differences. Though retaining small coal sites elevates extraction cost, these extra expenses are outweighed by carbon cost savings generated by phaseout of some high-emission large infrastructures.

We conduct a sensitivity analysis to test the robustness of results against deviations from the baseline fossil import share assumption (Figure S3). Across both scenarios, the results reveal narrow output variations under tested import shares, demonstrating that moderate shifts in fossil import shares exert marginal impacts on emissions, employment, and cost. This confirms that the impact gaps between the two retirement strategies remain robust regardless of import share adjustments. We systematically conducted sensitivity analysis for five key modeling assumptions (Figures S4–S8). Across all five groups of sensitivity simulations, the vertical error bars remain narrow relative to the absolute gap between STEPS and APS baseline outcomes, demonstrating that moderate perturbations to each single uncertain parameter only trigger limited quantitative deviations in national cumulative emission reduction, employment increase, and cost saving. Regardless of tested parameter bounds, two conclusions hold consistently: the STEPS scenario delivers larger emission cuts and employment gains under emission-oriented retirement, while the APS scenario often generates greater cost saving. Higher uncertainty emerges from perturbations to uncovered assets, yet even this parameter variation does not reverse the fact that emission reduction, employment increase, and cost saving can be achieved by emission-oriented strategy. The multiple sensitivity analysis verifies that our primary findings on the advantages of emission-oriented retirement strategy are robust against fluctuations of all key modeling assumptions.

## Discussion

The retirement strategy for small-scale fossil fuel facilities raises critical equity concerns in climate mitigation efforts. This approach allows large-scale, high-emission-intensity operations to maintain polluting practices while potentially eliminating smaller facilities that have adopted cleaner technologies. This regulation inadvertently encourages operators to scale up operations to avoid phaseout, potentially exacerbating overall emissions through capacity expansion. Conversely, the emission-oriented framework addresses inequities through a performance-based incentive mechanism, establishing clear benchmarks that encourage all facilities—regardless of size—to pursue continuous emission reductions and technological upgrades.

Policy switches impact groups in very different ways and existing stakeholders may resist policy implementation. Switching from the current strategy to the emission-oriented strategy enables a reduction in cumulative emissions in the country, increases in employment, and lower costs. The *emission-oriented* strategy would result in different spatial pattern of provincial fossil extraction compared to the *current* strategy (Figure S1). High-emission regions such as Shaanxi, Gansu, and Shandong experience cuts in cumulative emissions and employment, while energy supply regions such as Shanxi, Yunnan, and Inner Mongolia experience more emissions and employment due to expanded extraction. This requires a national-level regulation to coordinate emission-mitigation efforts across regions. First, it is essential to make the new strategy clear to all regions to form buy-in, and to provide an incentive for lowering emission intensity across regions.^27^^,^^28^ Second, in addition to pressure, financial support would also play an important role,^29^^,^^30^ especially for the industries that have the willingness and potential to reduce emissions but lack the investment to do so. Setting up a national-level fund could stimulate emission mitigation, especially in finance-scarce regions such as the northeast and southwest regions. Cross-provincial carbon benefit-sharing frameworks should be established to allocate nationwide emission reduction and employment retention benefits to regions bearing fossil early retirement cost. It is necessary to propose targeted vocational training and reemployment support for displaced fossil extraction workers to ease local employment decline for reconciling long-term national carbon neutrality targets with near-term regional economic stability.

Despite the high-coverage inventory constructed in this study, we cannot cover all coal, gas, and oil sites due to data availability. We assume the remaining sites continue to operate. We also do not include new sites that may be built in the future. In addition, the retirement strategy change would likely result in a reorganized fossil fuel transport network. The emissions from changed transport are often omitted in previous studies,^5^^,^^11^ including this study, given the small share (<3%)^31^^,^^32^^,^^33^ of transport in the life cycle emissions of fossil fuels in China. Different retirement sequences might redistribute fossil fuel output across provinces and reshape cross-regional fuel transportation routes. Variations in transport distance and carbon emissions of combustion fuels driven by shifted fuel flows are unaccounted for in combustion emission assessment. Such flow-related bias might affect combustion emissions between the two retirement strategies we compared. Follow-up research is suggested to couple our site-level extraction inventory with interprovincial freight transport models to map full fuel flow networks and quantify how reshaped fuel flows alter long-term combustion emissions under different retirement sequences. We examine the economic infeasibility of fossil sites driven by carbon price, but the economics of fossil use can also be driven by other factors (phase-out of fossil fuel finance, increased investment in labor, equipment, and transport, etc.)^34^^,^^35^ and decreased price of fossil fuels (due to globally variable energy, economic, and political situation, etc.).^36^ Compared with LCA studies that evaluate fixed asset footprints, our dynamic simulation further reveals long-term cumulative emission differences caused by different retirement sequencings; compared with MRIO research focusing on macro impacts, this study provides decision support for targeted micro asset-level fossil fuel infrastructure governance considering emissions, employment, and costs.

National policy is largely based on produced emissions from fossil fuel combustion but given that the fossil fuel extraction contributes 15% of total emissions these should not be ignored. Considering both extraction and combustion emissions reveals a greater emission reduction potential through emission-oriented asset retirement sequencing than extraction-focused studies.^11^ In addition, China’s official database provides national-average emission intensities of coal extraction, but not oil and gas extraction^37^ even though their contributions are non-negligible, as we show here. Given China’s insufficient domestic supply of oil and gas, it is unlikely that these extraction systems will phase out soon, but the rapid advancement of renewables and energy storage technologies may provide an extra pressure. If policy makers were to follow our analysis here the result would mean fewer cumulative emissions, the maintenance of jobs for longer, and reduced costs. However, regional policies would still need to be in place to provide support for areas where assets are retired.^38^

### Limitations of the study

Several limitations of this study should be acknowledged. First, emission intensities are treated as static throughout the 2024–2050 period. In practice, technological improvements—such as methane capture, electrification of mining equipment, and efficiency gains—could reduce extraction-stage emission intensities over time, which would affect the relative ranking of assets and the magnitude of sequencing benefits. Second, the share of fossil fuels imported into China is held constant at 2023 levels (7% for coal, 75% for oil, 40% for gas) throughout the projection period. This assumption may not fully reflect China’s evolving energy security objectives or geopolitical dynamics, and alternative import trajectories could shift the domestic production volumes and the composition of the retirement queue. Third, our analysis does not distinguish between coking coal (used primarily in steelmaking) and thermal coal (used primarily for power generation). These two coal types face different demand trajectories and substitution constraints; applying a uniform sequencing rule across both may overstate the feasibility of retiring certain high-intensity coking coal mines. Where data permit, future work should treat these separately. Fourth, the combustion fraction parameter θ is applied at the national level and does not vary across sites or over time. As China’s coal-to-chemicals and petrochemical sectors expand, the non-combustion share of fossil fuel use may increase, which would reduce combustion emission estimates. Fifth, the carbon price trajectory used to assess economic infeasibility is an illustrative scenario from IEA World Energy Outlook (WEO) 2024 and does not represent current Chinese policy. Results are sensitive to the assumed carbon price level and timing, and should be interpreted as indicative of the direction and order of magnitude of sequencing benefits rather than precise forecasts. Existing studies have proven the effective application of artificial intelligence approaches including neural networks in thermodynamic calculation and energy system modeling. In follow-up extensions of this work, our asset-level simulation framework for fossil fuel extraction infrastructure retirement can be integrated with such artificial intelligence techniques. For example, neural networks can be deployed to dynamically predict time-varying emission intensities of mines and oil/gas fields driven by technical progress; AI-based multi-objective optimization algorithms can replace static sequencing rules to jointly optimize carbon abatement, job retention, and system costs.

## Resource availability

### Lead contact

Further information and requests for resources should be directed to and will be fulfilled by the lead contact, Xiaoyang Zhong (x.zhong@sz.tsinghua.edu.cn).

### Materials availability

This study did not generate new materials.

### Data and code availability

We provide the complete plant-by-plant dataset for coal, oil, and gas extraction sites, with variables on location, emissions, employment, and cost, archived at Zenodo: https://doi.org/10.5281/zenodo.22194981. The scripts used to calculate emissions, employment, and costs, and to generate the figures, are available on github: https://github.com/tcjy1204/fossil-retirement.

## Acknowledgments

This work was supported by The National Social Science Fund of China (grant no. 25FTJB008) and major program of National Social Science Fund of China (grant no. 25&ZD140). X.Z. was supported by the start-up fund of 10.13039/100018913Tsinghua Shenzhen International Graduate School, 10.13039/501100004147Tsinghua University.

## Author contributions

Conceptualization, Y.J. and X.Z.; data curation, Y.J., X.T., and Z.M.; software, Y.J.; methodology, Y.J., X.Z., and P.B.; investigation, Y.J., X.Z., Y.C., and P.B.; visualization, Y.J.; writing – original draft and writing – review and editing, Y.J., X.Z., A.T., E.B., W.L., L.T., B.v.R., and P.B.

## Declaration of interests

The authors declare no competing interests.

## STAR★Methods

### Key resources table

**Table:** 

REAGENT or RESOURCE	SOURCE	IDENTIFIER
**Deposited data**		

Coal mines	China National Coal Association; Global Energy Monitor	https://www.coalchina.org.cn/ https://globalenergymonitor.org/
Oil and gas fields	China Petrochemical Corporation; China National Petroleum Corporation; China National Offshore Oil Corporation; IHS Markit;	https://www.sinopec.com/ https://www.cnpc.com.cn/cnpc/index.shtml https://www.cnooc.com.cn/ https://vantage.ihsenergy.com
Fossil fuel demand of China	World Energy Outlook 2024	https://www.iea.org/reports/world-energy-outlook-2024

**Software and algorithms**		

MATLAB software	MathWorks	https://ww2.mathworks.cn/

### Experimental model and study participant details

No experimental models or study participants are used in this study.

### Method details

#### Overview

This study first constructs a high-resolution spatiotemporal micro-inventory of China’s fossil fuel extraction infrastructure by integrating multiple authoritative datasets from industry associations, literature and major domestic oil corporations. The inventory covers coal mines, oil fields and gas fields, containing detailed attributes including geographic location, annual output, remaining reserves, extraction cost and on-site employment. We apply a greenhouse gas accounting framework to calculate CO_2_-equivalent emissions based on Global Warming Potential indicator (GWP) for each site. The total emissions of each asset consist of two parts: extraction emissions and combustion emissions determined by site-specific fuel carbon content, heating value, oxidation rate and non-combustion utilization ratio. We unify the two parts into a total emission intensity formula for every single extraction site, which is the sorting indicator of the emission-oriented retirement strategy in this study. We design a dynamic retirement simulation framework covering 2024–2050, matching two IEA energy demand scenarios with two distinct infrastructure retirement sequencing strategies to form four retirement scenarios. The asset retirement follows a hierarchical logic: reserves-depleted assets and economically unviable assets under carbon price pressure will be retired first; to meet the annual domestic fossil supply reduction target, additional capacity will be phased out under two separate rules, namely the current strategy that prioritizes small-scale sites and the emission-oriented strategy. For each year, we sort assets by corresponding rules, close high-priority sites and proportionally cut output of marginal assets to match the required annual capacity reduction volume. We make a three-dimensional quantitative evaluation to compare the climate, social and economic differences between the two strategies. From the climate dimension, we calculate annual and cumulative CO_2_e emissions of all operating sites to quantify the emission reduction potential brought by emission-oriented strategy. From the social dimension, we compute retained full-time equivalent job-years. From the economic dimension, we calculate the net present value of total costs, including extraction and carbon cost. Other components (e.g., transport, ancillary services) may be sequencing-responsive, but their contributions are expected to be second-order and are commonly omitted in prior work.^5^^,^^11^ Results should be interpreted as the direct effects of retirement sequencing on the dominant components. Figure 3 overviews the analytical workflow; subsequent sections describe the data, retirement strategies, and quantification of the impacts.

#### Fossil fuel information

##### Fossil fuel extraction

The coal mine dataset is sourced from the China National Coal Association^39^ and Global Energy Monitor,^40^ which includes the information on mine name, annual output, location, start year, remaining reserve, operating status, mine depth, mine type, employment and extraction cost. We filter them for remaining operating mines in 2023. We include 1265 operating coal mines with a total annual output of 3,762 million tons.^41^ There are 1109 underground mines (2,837 million tons) and 156 surface mines (925 million tons). The dataset of oil and gas fields is obtained from IHS Markit^42^ and China’s three major petroleum corporations, i.e., the China National Petroleum Corporation (CNPC), the China Petrochemical Corporation (SINOPEC), and the China National Offshore Oil Corporation (CNOOC).^43^^,^^44^^,^^45^ This dataset includes data on: field name, annual output, start year, remaining reserve, operating status, location, field type, employment and extraction cost. We include 841 operating oil fields and 587 operating gas fields in 2023, accounting for 182.5 Mt of oil extraction and 175.7 Gm^3^ of gas extraction. Our inventory covers 81%, 82%, and 89% of national coal, gas, and oil output respectively, while the remaining unrecorded small-scale scattered extraction sites are assumed to maintain fixed annual output throughout 2024–2050. This is a relatively restrictive assumption mainly caused by data access barriers: small private mines and scattered oil/gas wells lack standardized public statistics on reserves, emission intensity, employment and extraction costs, making asset-by-asset dynamic simulation infeasible for these units. Given their relatively low share of national output and the relatively long national-average remaining production time of existing coal mines (35 years), oil fields (24 years), and gas fields (33 years), fixing their total volume simplifies the modeling framework without fundamentally changing the comparative gap between the two retirement sequencing strategies we focus on. Nevertheless, this simplification introduces uncertainty, as some sites may phase down due to reserve depletion and negative profit over the period. Future research can integrate more micro-statistics to expand inventory coverage to relax this constant-production hypothesis. A sensitivity analysis has been conducted to assess the impacts of uncovered assets on emissions, employment, and cost.

##### Fossil fuel demand

The fossil fuel demand of China is obtained from the World Energy Outlook 2024,^26^ which presents the demand (*df*) for coal, oil, and gas for the years 2023, 2030, 2035, and 2050 under two scenarios, i.e., the Stated Policies Scenario (STEPS) and the Announced Pledges Scenario (APS). STEPS provides a sense of the energy sector’s direction of travel today, based on the latest market data, technology costs and in-depth analysis of the prevailing policy settings in countries around the world. The APS examines what would happen if all national energy and climate targets made by governments are met in full and on time. Referring to Liu et al. (2024),^11^ a constant proportion (*pi*) of imported fossil fuels is applied to the future situation, which is 7%, 75%, and 40% for coal, oil, and gas, respectively. Then, we obtain the annual demand for domestic fossil fuels (*d*_*t*_) by multiplying *df* by (1-*pi*). *d*_*t*_ is further interpolated with a least-square curve between 2023 and 2050.

##### Emission intensity

We calculate greenhouse gas (GHG) emissions from both extraction and combustion. Two major types of greenhouse gas are considered, i.e., CO_2_ and CH_4_, and results are expressed as CO_2_-equivalents (CO_2_e) using 100-year global warming potentials (GWP100).^46^ The emission intensity of coal extraction is obtained from multiple sources,^47^^,^^48^^,^^49^^,^^50^^,^^51^ covering the CO_2_ and CH_4_ emissions from mining and post-mining for coal (including fugitive emissions and the emissions from energy use). We use heating values and net carbon content of coal from Liu et al. (2015),^16^ which presents the heating values and net carbon content at the mine level based on site-specific coal samples in China. We match these values with coal mines in our inventory. In addition, we assess the provincial-level coal heating values and net carbon content according to Liu et al. (2015) and apply them to the mines that are not included in that study based on the location of those mines. The emission intensity of oil and gas is assessed based on multiple sample data sources. Specifically, the emission intensity of oil and gas extraction is sourced from Tang et al.^52^ The emission intensity of oil and gas combustion is assessed based on the data on oil and gas from Liu et al.^53^ and Xu et al.,^54^ which measured the proportion of the mass of carbon in oil and gas at the level of oil and gas fields. For coal, oil, and gas, the emission intensity is assessed according to emission accounting method^55^^,^^56^^,^^57^ as below:

(Equation 1) $${tei}_{i}={pei}_{i}+{cei}_{i}={pei}_{i}+{ef}_{i}\times \left(1-\theta \right)\times \eta$$

where, *tei*_*i*_ represents the emission intensity of the fossil fuel from site *i*, *pei*_*i*_ the extraction emission intensity*, cei*_*i*_ the combustion emission intensity, *ef*_*i*_ the emission factor of combustion of fossil fuels from site *i*, *θ* the proportion of fossil fuels that are not burnt, *η* the oxidization rate.

#### Fossil fuel retirement strategies

##### Annual output reduction

The quantity of fossil fuels (*q*_*t*_) in our inventory that needs to be reduced in year *t* is the extraction in 2023 of our inventory (*p*_2023_) minus the demand for domestic fossil fuels in year *t* (*d*_*t*_). Our fossil fuel extraction dataset covers about 80% of the national totals due to data limitations. The excluded ∼20% (*p*_*ex*_) is assumed to exist constantly in the future.

(Equation 2) $$q_{t}=p2023+p_{ex}-d_{t}$$

##### Drivers of retirement

Fossil fuel sites typically close when reserves are depleted, extraction becomes economically infeasible, or regulations mandate their shutdown. We use a decision-making flow (Figure 4) to determine the order of fossil-fuel site closure according to national policies and research.^58^^,^^59^^,^^60^

First, a fossil fuel site has to be closed when it runs out of its remaining reserve, which is referred to as reserve depletion. Here, the remaining time of each fossil fuel site is obtained by dividing its remaining reserve by the output in 2023, with an assumption that each site generates the same amount of fossil fuel annually before closure.

A carbon market is also emerging in China with the aim of driving emissions down.^61^ We incorporate carbon cost here whereby fossil fuel sites operate when fossil fuel price is higher than cost (extraction cost plus carbon price) but would not when fossil fuel price is lower than cost. The economic-feasibility status of each site is assessed considering both price and cost of fossil fuel production^38^^,^^62^ as follows:

(Equation 3) $$I=p-c-t_{c}$$

where, *I* refers to the profit of coal mine/oil field/gas field (CNY). A site is retired by carbon price when *I* < 0. *p* denotes the price of coal mine/oil field/gas field (CNY/t for coal and oil, CNY/m^3^ for gas), *c* the extraction cost of coal mine/oil field/gas field (CNY/t for coal and oil, CNY/m^3^ for gas), *t*_*c*_ the carbon price (CNY/t). For the implementation timeline, carbon price pressure only applies to extraction sites starting from 2030 in both scenarios while no carbon cost is imposed on extraction facilities during 2024–2029. In terms of price, APS maintains persistently higher carbon prices than STEPS after 2030, driven by more ambitious emission reduction commitments. There are differences between the modeled IEA carbon price framework and China’s current operational carbon market. In sectoral coverage, domestic ETS only regulates power combustion emissions, while the hypothetical carbon price covers all extraction and downstream combustion emissions of fossil fuels. In price formation, China’s emission allowance price is determined by quota supply and demand, whereas IEA carbon prices are derived from global marginal abatement cost with higher long-run levels under APS. In policy attribute, domestic carbon prices are enforceable regulatory instruments, while the IEA price series are analytical assumptions for scenario testing.

It is important to note that China has not implemented a carbon tax on fossil fuel extraction. China’s national Emissions Trading Scheme (ETS) currently covers the power generation sector and does not extend to upstream extraction activities. The carbon price incorporated in our model is a forward-looking scenario assumption drawn from the IEA World Energy Outlook 2024, which projects a carbon price trajectory beginning in 2030 under both STEPS and APS. This is used as a policy stress test to assess the economic feasibility of extraction sites under plausible future carbon pricing conditions, consistent with standard practice in long-run decarbonisation analysis. It should not be interpreted as a description of current Chinese policy.

When the output reduction driven by reserve depletion and economic infeasibility is less than the quantity of output that needs to be reduced, additional fossil fuel sites will be forced to close by regulations. Here, two regulation strategies (*current* strategy and *emission-oriented* strategy) are examined, with *current* strategy prioritising shutting down smaller sites and the *emission-oriented* strategy prioritising high-emission-intensity sites.

Further, some other important factors have been considered in determining the closure sequence of fossil fuel sites. Specifically, safety issues are important for coal mining. Methane concentration usually increases with the depth of mines,^63^^,^^64^ and deep mines are considered unsafe compared to shallow mines. Deeper mines have a higher prioritisation for closure than shallow mines. There are no policies to force the closure of oil and gas fields, largely because China heavily relies on the two resources. Referring to the literature, cost is a major driver of oil and gas extraction^65^ and is used as a sorting criterion of oil and gas fields for closure, i.e., higher-cost fields have a higher prioritisation for closure than lower-cost fields.

A combination of two energy demand scenarios (STEPS, APS) and two fossil fuel closure strategies (*current*, *emission-oriented*) generates four fossil fuel site closure scenarios: STEPS-*current*, STEPS *emission-oriented*, APS-*current*, APS-*emission-oriented* (Table 1). For each scenario, we define a series of fossil fuel sites: *X*_*1*_ … *X*_*n*_ *X*_*n*+1_ *X*_*n*+1_ … *X*_*m*_

We define *q*_*i*_ as the output of fossil fuel site *X*_*i*_. *q*_*t*_ the quantity of fossil fuels in our inventory that needs to be reduced in year *t.* Now if $\sum_{1}^{n}q_{i}\leq q_{t}<\sum_{1}^{n+1}q_{i}$ we close sites 1∼*n*, while site (*n*+1)∼*m* keeps operating, and site (*n*+1) sees an output reduction of $q_{t}-\sum_{1}^{n}q_{i}$.

#### Emissions implications

Extraction contributes to system-wide emissions via two channels: (i) direct, on-site energy use and fugitive releases, and (ii) downstream combustion governed by site-specific carbon content and heating value. For each energy-transition scenario, we calculate annual on-site and combustion emissions under two retirement sequences—the status quo (retiring smaller sites first) and an alternative that prioritizes high-emission-intensity sites—and compare cross-strategy differences to quantify the implications of sequencing.

##### Emission estimate

The total emission of the remaining operating infrastructures is assessed as follows:

(Equation 4) $$em=\sum_{1}^{n+1}{em}_{i}=\sum_{n+1}^{m}q_{i}\cdot {ei}_{i}+\left(\sum_{1}^{n+1}q_{i}-q_{t}\right)\cdot {ei}_{n+1}$$

where *em* is annual emission from extraction and subsequent combustion, assessed by multiplying the output of each fossil fuel site by the corresponding emission intensity (*ei*_*i*_). The emission intensity is defined as the total carbon emissions per unit of fuel output (tCO_2_-eq/TJ), calculated as the sum of contributions from extraction and combustion. As mentioned above, *q*_*i*_ refers to the output of fossil fuel site *X*_*i*_. *q*_*t*_ the quantity of fossil fuels in our inventory that needs to be reduced in year *t*.

#### Employment and cost implications

After identifying the lowest-emissions sequence, we evaluate its employment and extraction-cost implications. Specifically, we compute annual extraction-stage employment and costs for each scenario and retirement sequence, then compare the differences between the lowest-emissions sequence and the status-quo strategy. Employment and costs are evaluated only at the extraction stage, as retirement sequencing does not materially affect downstream combustion jobs or costs.^66^^,^^67^

##### Employment estimate

The employment of people in the remaining operating infrastructures is assessed as follows:

(Equation 5) $$emm=\sum_{1}^{n+1}{emm}_{i}=\sum_{n+1}^{m}q_{i}\cdot {emi}_{i}+\left(\sum_{1}^{n+1}q_{i}-q_{t}\right)\cdot {emi}_{n+1}$$

where *emm* is annual employment, which is assessed by multiplying the output of each fossil fuel site by the corresponding employment intensity (*emi*_*i*_). Here, *emi*_*i*_ is assessed by dividing the employment by the output of each site.

##### Cost estimate

The cost of the remaining operating infrastructures is assessed as follows:

(Equation 6) $$c=\sum_{1}^{n+1}c_{i}=\sum_{n+1}^{m}q_{i}\cdot {ci}_{i}+\left(\sum_{1}^{n+1}q_{i}-q_{t}\right)\cdot {ci}_{n+1}$$

where *c* is annual cost, which is assessed by multiplying the output of each fossil fuel site by the corresponding cost per unit of fossil fuel (*ci*_*i*_). Here, both the extraction cost and the carbon cost driven by carbon price are considered. The carbon price is sourced from International Energy Agency for scenario STEPS and APS. The carbon price is lower in STEPS than in APS (Data S1).

#### Sensitivity and uncertainty analysis

Given the fixed import share of coal, oil and natural gas (7%, 75% and 40% respectively) assumed in this study, we construct a targeted sensitivity analysis to quantify how changes in fossil fuel import shares alter modeling outputs. We set bounded variation ranges for each fuel’s import share: coal import share ranging from 4% to 7%, oil from 72% to 78%, and natural gas from 37% to 43%. The model is run for each import share while keeping others unchanged, i.e., one-at-a-time method is applied here for sensitivity analysis. For parameters including carbon prices, fuel prices, emission factors and non-combustion fuel shares, we impose ±10% bounded perturbations relative to our benchmark; for uncovered fossil assets, we test two boundary cases where unregulated facilities inherit either the lowest or highest emission intensity profiles of existing infrastructure. All sensitivity figures separate cumulative emission reduction, employment increase and total cost saving between the STEPS and APS scenarios, with vertical error bars illustrating the full range of fluctuations induced by parameter perturbations.

### Quantification and statistical analysis

All quantitative analyses and simulations were performed using MATLAB.^68^ Annual fossil fuel demand between the reported IEA scenario years was interpolated using least-squares curve fitting. The subsequent asset-level retirement and impact analyses are deterministic, and no inferential statistical tests were performed. For site-level analyses, *n* denotes the number of fossil fuel extraction infrastructures included; the inventory comprises 2,693 operating sites, including 1,265 coal mines, 841 oil fields, and 587 gas fields. Sensitivity analyses were conducted using one-at-a-time parameter perturbations as described above. Error bars in the sensitivity analyses represent the full range of model outcomes across the tested parameter bounds rather than confidence intervals or other measures of statistical uncertainty.

## Supplemental information

Document S1. Figures S1–S8 and Table S1

Data S1. Asset-level output, emissions, employment, costs, and scenario parameters for fossil fuel extraction infrastructure during 2024–2050
