# Supplementary material for: Large emission mitigation potential from sequencing fossil fuel extraction infrastructure retirement in China
Source: iScience. 2026 Sep 18;29(10):117563. doi: 10.1016/j.isci.2026.117563 (PMC13627143; doi:10.1016/j.isci.2026.117563)
Supplement: Supplementary file 1 [file mmc1.pdf]

iScience, Volume *29*

Supplemental information

Large emission mitigation potential from sequencing fossil fuel extraction infrastructure retirement in China

Yi Jin (金艺), Xiaoyang Zhong (钟晓阳), Arnold Tukker, Xu Tang, Elina Brutschin, Zhida Ma, Yingchao Chen, Wenchao Li, Lixin Tian, Bas van Ruijven, and Paul Behrens

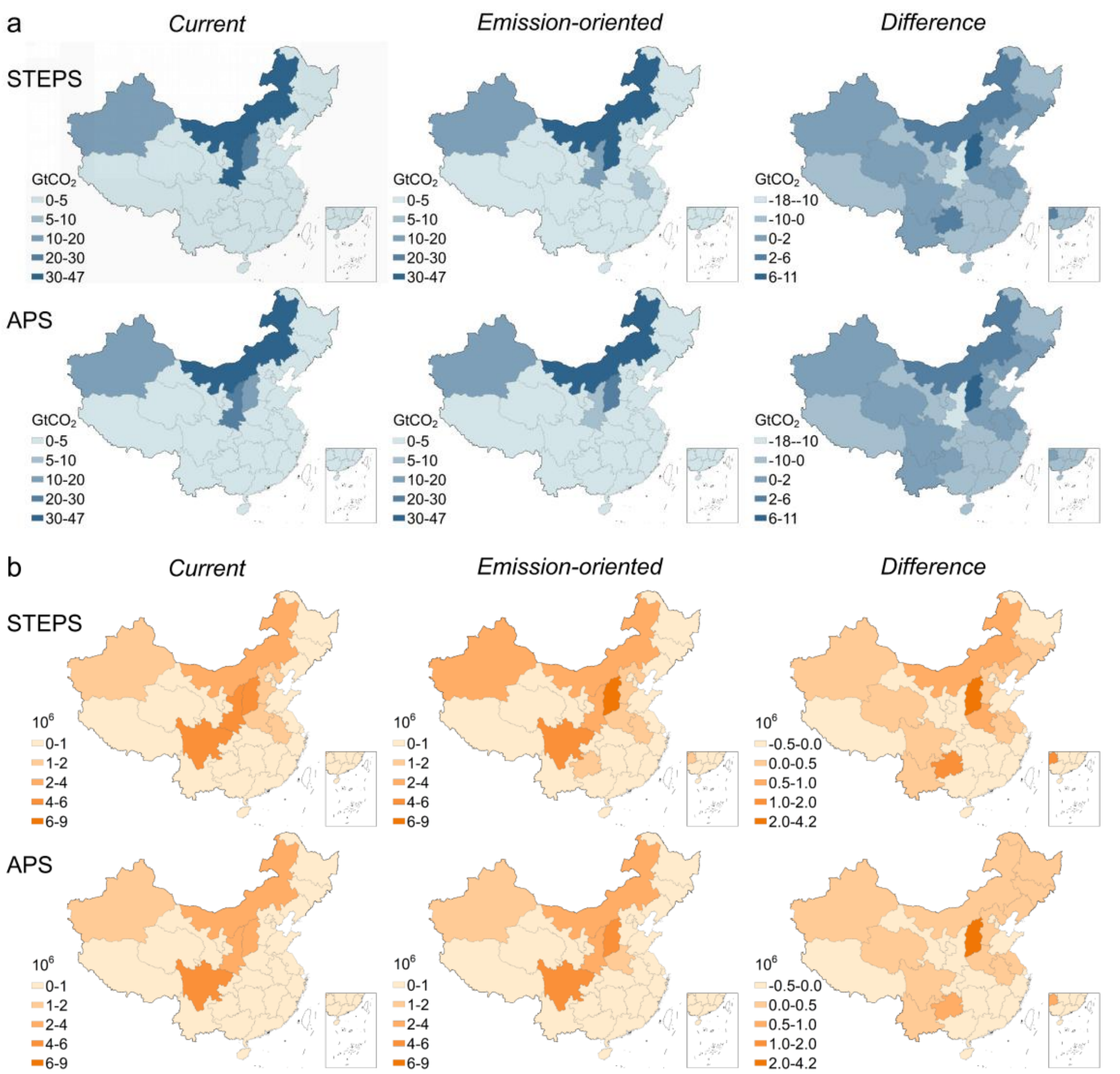

Fig. S1| Provincial-level cumulative emissions and employment over the period 2024-2050 under the *current* and *emission-oriented* strategies and the difference between the two strategies. a, emissions; b, employment.

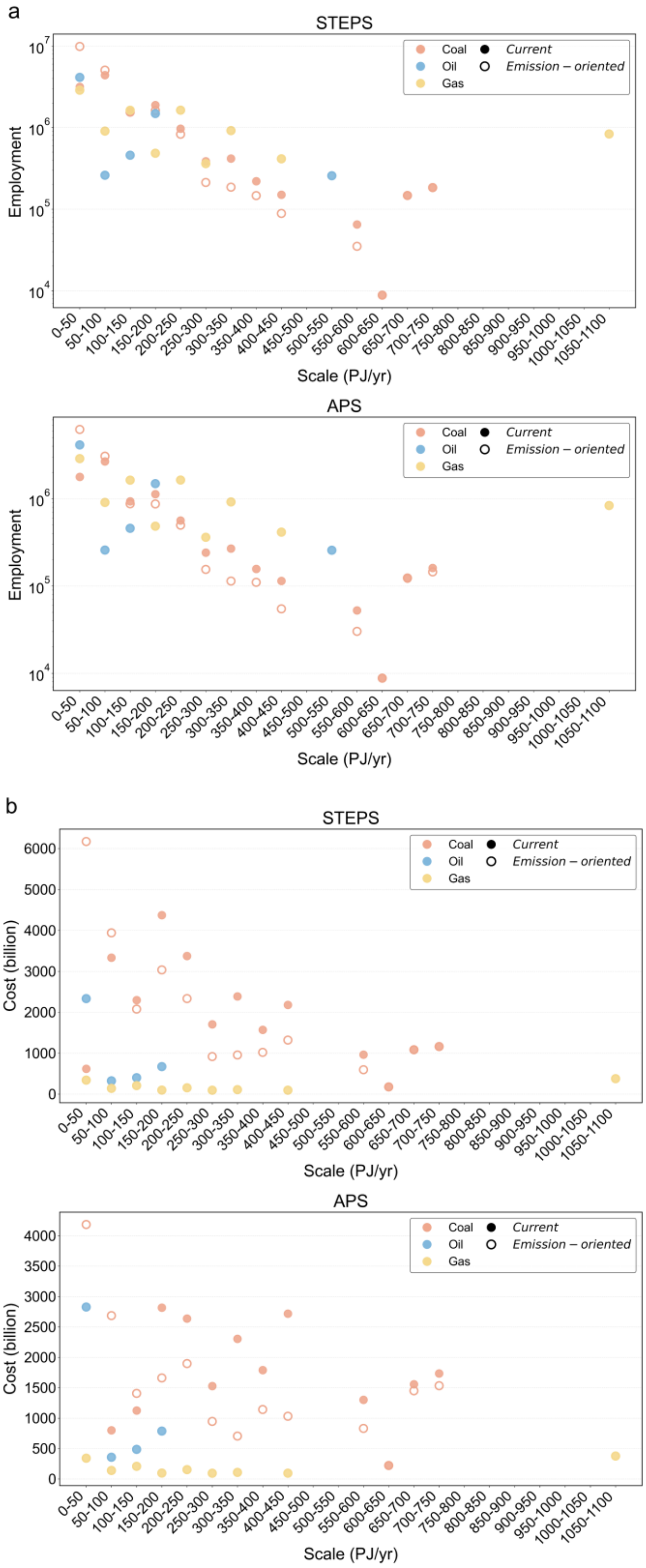

Fig. S2| Cumulative employment and overall cost against annual facility scale (PJ yr^{-1}) across STEPS and APS scenarios, distinguishing coal, oil and gas. Colour coding represents fuel type; solid markers denote outcomes under the *current* strategy while hollow markers represent the *emission-oriented* strategy.

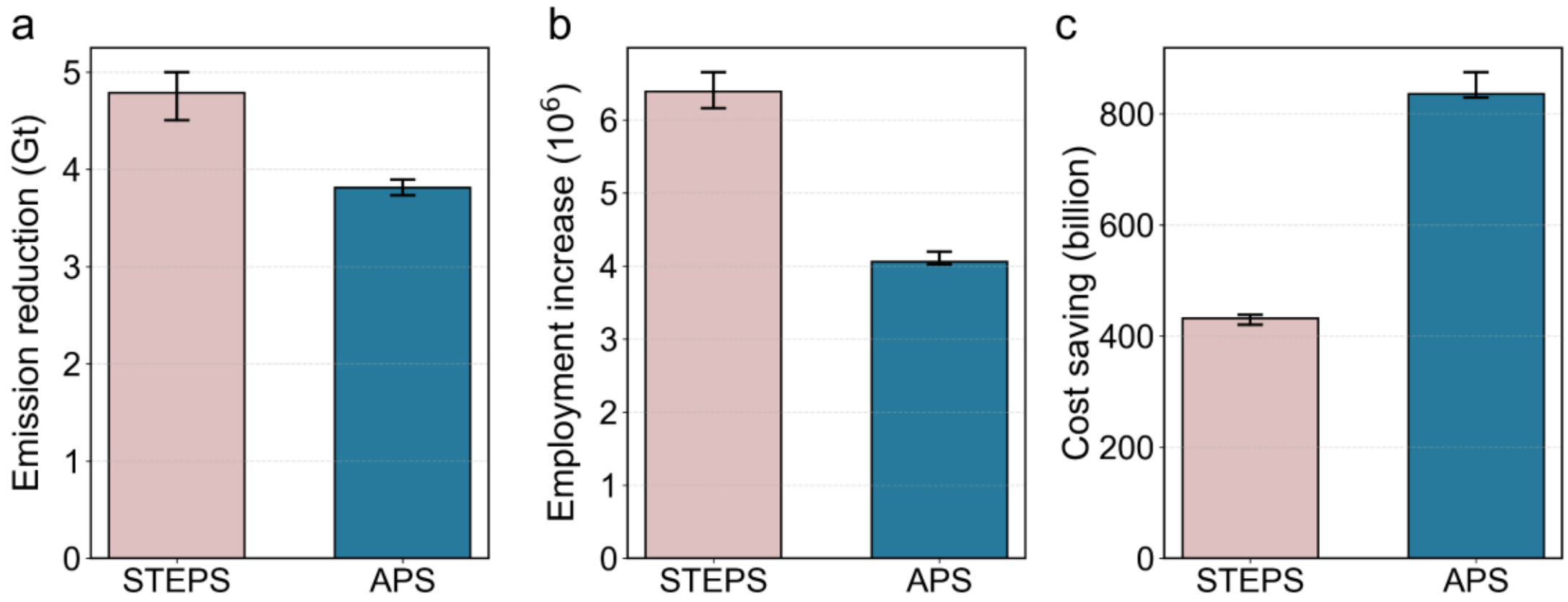

Fig. S3| Cumulative national emissions, employment and cost under different levels of import shares of coal, oil, and gas. The bars represent emissions, employment and cost with current import shares, i.e., 7% for coal, 75% for oil, and 40% for natural gas, while error bars represent emissions, employment and cost with new tested import shares 4%-7% for coal, 72%-78% for oil, and 37%-43% for natural gas.

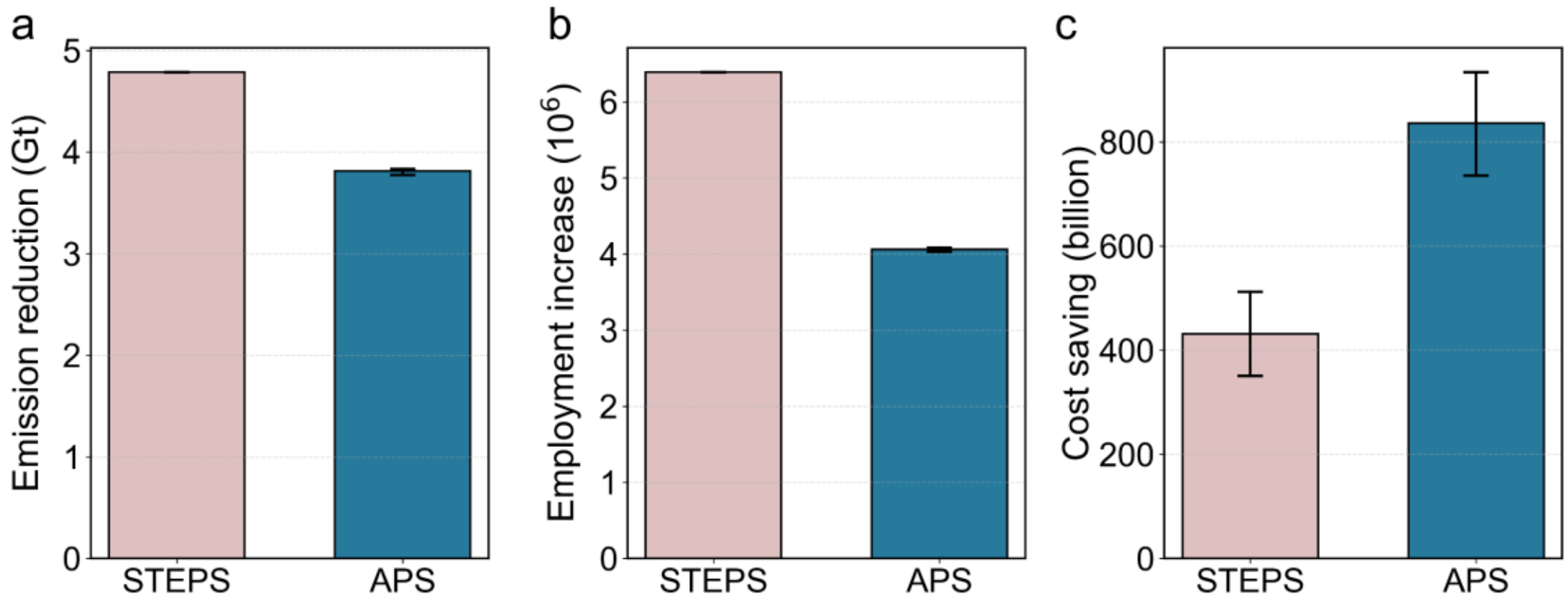

Fig. S4| Cumulative national emissions, employment and cost under varied carbon price levels (±10% relative to the model benchmark).

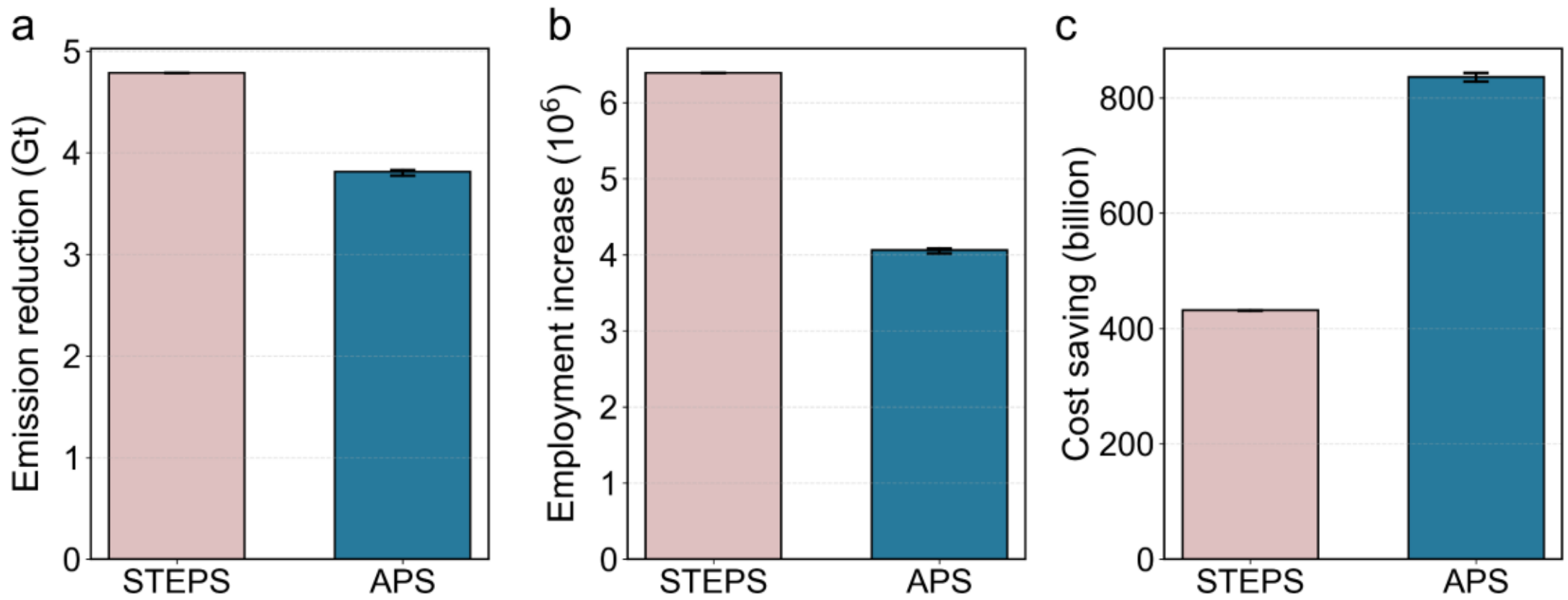

Fig. S5| Cumulative national emissions, employment and cost under varied coal, oil and gas fuel price levels (±10% relative to the model benchmark).

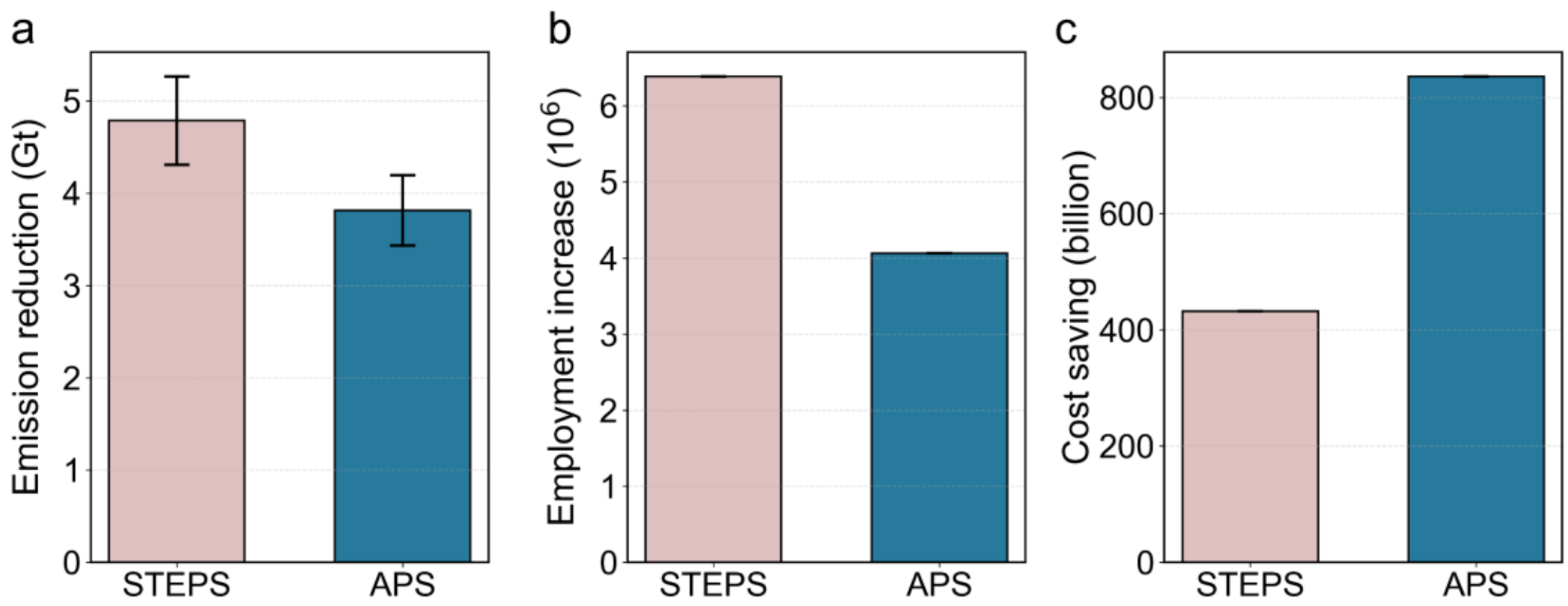

Fig. S6| Cumulative national emissions, employment and cost under varied emission factor levels for coal, oil and gas (±10% relative to the model benchmark).

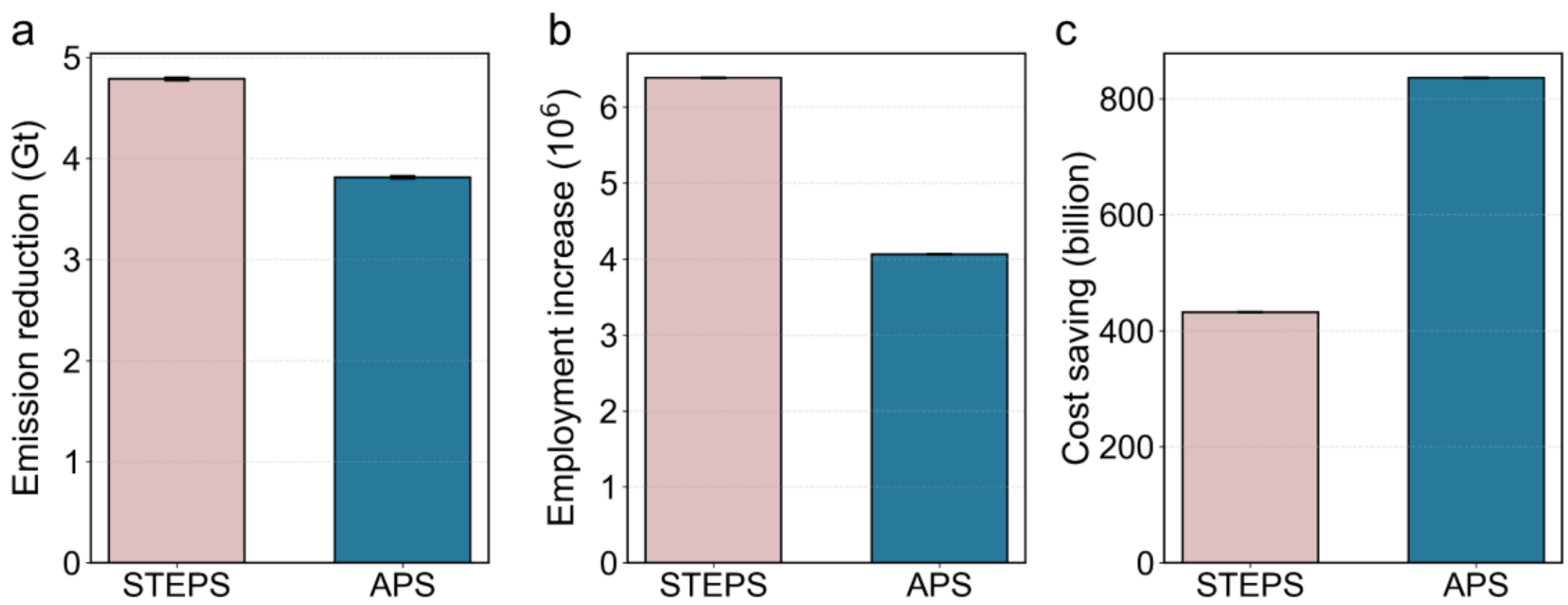

Fig. S7| Cumulative national emissions, employment and cost across varied shares of non-combustion fossil fuel utilisation (±10% relative to the model benchmark).

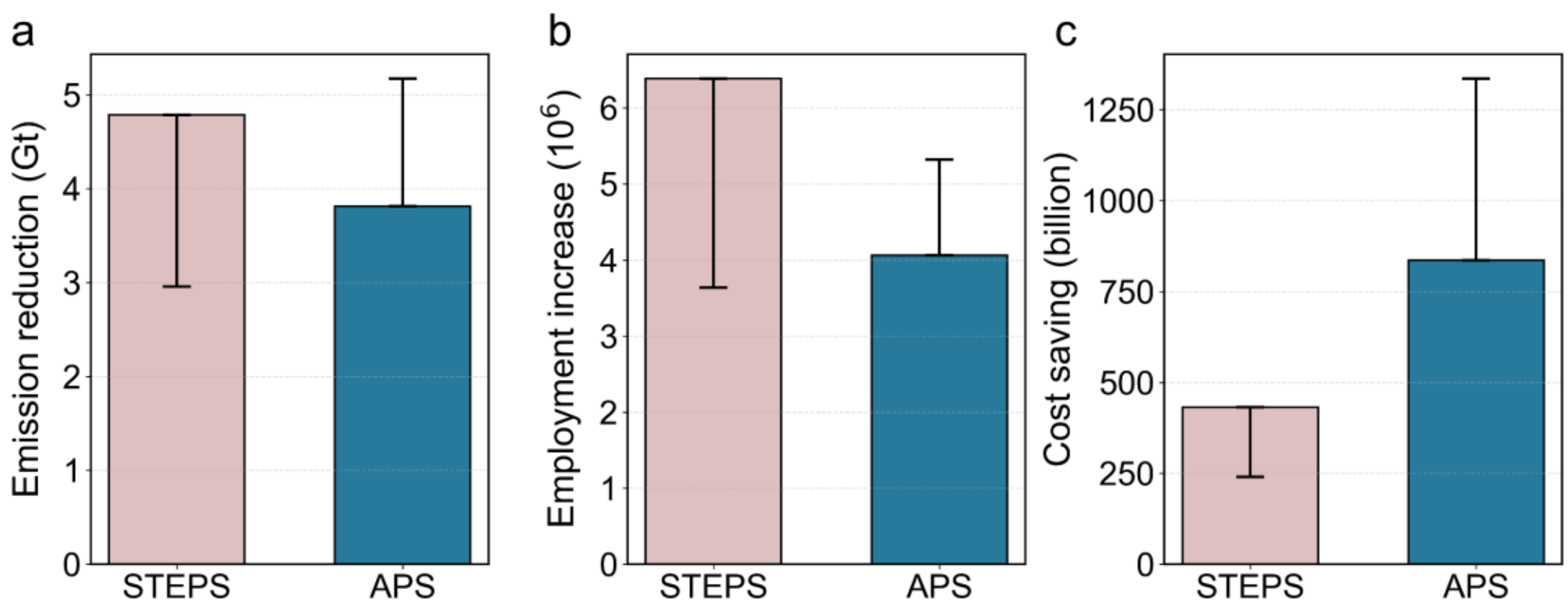

Fig. S8| Cumulative national emissions, employment and cost under different characteristics of uncovered fossil fuel assets (uncovered assets are assigned the characteristics of either the lowest-emission-intensity assets or the highest-emission-intensity assets).

Table S1 Comparison with previous studies.

**Study**	**Scope**	**Resolution**	**Sector**	**Retirement logic**	**Key metrics**
Liu et al. (2024)[1]	Coal mine methane	Asset-level: coal mines	Coal	Prioritise high-gas-content mines	Cumulative methane emission reduction
Zhang et al.[2]	Downstream power carbon emissions	Power units	Coal power sector	Age, cumulative emissions, carbon quota	Stranded value of coal power
Wang et al.[3]	Downstream power carbon emissions	Power units	Coal power sector	Cost and benefit	Stranded value of coal power
Zhang et al.[4]	Downstream power carbon emissions	Power units	Coal power sector	Years of service since construction, capacity size, energy efffciency, technology type, and ownership	Stranded value of coal power
Semieniuk et al.[5]	oil and gas asset	Asset-level: oil fields, gas fields	oil and gas	Cost and benefit	Profit losses
Li et al.[6]	Downstream power carbon emissions	Power units	Coal power sector	Technological, profitability, and environmental impacts	Stranded value of coal power
Navarro et al.[7]	Downstream power carbon emissions	Power units	Fossil-fired power plants	Profitability	Stranded value of fossil-fired power
This study	Upstream extraction emissions and downstream combustion emissions	Asset-level: coal mines, oil fields, gas fields	Coal, oil, gas	Remaining production time, cost and benefit, two sequencing strategies: 1) *current*: phaseout small sites first; 2) *emission-oriented*: phaseout high emission-intensity sites first	Cumulative emissions, retained employment, cost

References

1. Liu, Q., Teng, F., Nielsen, C.P., Zhang, Y. & Wu, L., Large methane mitigation potential through prioritized closure of gas-rich coal mines. *Nature Climate Change* **14**,6, 652-658(2024). 10.1038/s41558-024-02004-3
2. Zhang, w. & Yuan, j., China's coal power sector stranded assets under the 2℃ global carbon constraint. *CLIMATE CHANGE RESEARCH* **17**,01, 36-44(2021).
3. Wang, Y., Wang, K., Liu, J. & Zou, J., China's coal power stranded assets under carbon lock-in constraint. *China Environmental Science* **42**,03, 1427-1434(2022). 10.19674/j.cnki.issn1000-6923.20211022.003
4. Zhang, W., Ren, M., Kang, J., Zhou, Y. & Yuan, J., Estimating stranded coal assets in China's power sector. *Utilities Policy* **75**, 101352(2022). https://doi.org/10.1016/j.jup.2022.101352
5. Semieniuk, G., et al., Stranded fossil-fuel assets translate to major losses for investors in advanced economies. *Nature Climate Change* **12**,6, 532-538(2022). 10.1038/s41558-022-01356-y
6. Li, B., et al., Projection of stranded coal power assets in China under the 1.5 °C global warming level. *Applied Energy* **416**, 127940(2026). https://doi.org/10.1016/j.apenergy.2026.127940
7. Fofrich Navarro, R., Liebermann, L., Moore, F.C., Shearer, C. & Davis, S.J., Ownership of power plants stranded by climate mitigation. *Nature Sustainability* **9**,2, 328-336(2026). 10.1038/s41893-025-01707-5
